# Zinc-Finger Antiviral Protein Inhibits XMRV Infection

**DOI:** 10.1371/journal.pone.0039159

**Published:** 2012-06-15

**Authors:** Xinlu Wang, Fan Tu, Yiping Zhu, Guangxia Gao

**Affiliations:** 1 Key Laboratory of Infection and Immunity, Institute of Biophysics, Chinese Academy of Sciences, Beijing, China; 2 Graduate School of Chinese Academy of Sciences, Beijing, China; National Institute of Allergy and Infectious Diseases – Rocky Mountain Laboratories, United States of America

## Abstract

**Background:**

The zinc-finger antiviral protein (ZAP) is a host factor that specifically inhibits the replication of certain viruses, including Moloney murine leukemia virus (MoMLV), HIV-1, and certain alphaviruses and filoviruses. ZAP binds to specific viral mRNAs and recruits cellular mRNA degradation machinery to degrade the target RNA. The common features of ZAP-responsive RNA sequences remain elusive and thus whether a virus is susceptible to ZAP can only be determined experimentally. Xenotropic murine leukemia virus-related virus (XMRV) is a recently identified γ-retrovirus that was originally thought to be involved in prostate cancer and chronic fatigue syndrome but recently proved to be a laboratory artefact. Nonetheless, XMRV as a new retrovirus has been extensively studied. Since XMRV and MoMLV share only 67.9% sequence identity in the 3′UTRs, which is the target sequence of ZAP in MoMLV, whether XMRV is susceptible to ZAP remains to be determined.

**Findings:**

We constructed an XMRV-luc vector, in which the coding sequences of Gag-Pol and part of Env were replaced with luciferase-coding sequence. Overexpression of ZAP potently inhibited the expression of XMRV-luc in a ZAP expression-level-dependent manner, while downregulation of endogenous ZAP rendered cells more sensitive to infection. Furthermore, ZAP inhibited the spreading of replication-competent XMRV. Consistent with the previously reported mechanisms by which ZAP inhibits viral infection, ZAP significantly inhibited the accumulation of XMRV-luc mRNA in the cytoplasm. The ZAP-responsive element in XMRV mRNA was mapped to the 3′UTR.

**Conclusions:**

ZAP inhibits XMRV replication by preventing the accumulation of viral mRNA in the cytoplasm. Documentation of ZAP inhibiting XMRV helps to broaden the spectrum of ZAP's antiviral activity. Comparison of the target sequences of ZAP in XMRV and MoMLV helps to better understand the features of ZAP-responsive elements.

## Introduction

The zinc-finger antiviral protein (ZAP) was initially recovered as a host factor that inhibits Moloney murine leukemia virus (MoMLV) infection [1]. In addition to MoMLV, ZAP inhibits the replication of HIV-1, certain alphaviruses and filoviruses [2], [3], [4]. However, ZAP does not induce a universal antiviral state because some viruses replicate normally in ZAP-expressing cells [2].

Analyses for the step at which ZAP blocks MoMLV replication reveal that ZAP prevents viral mRNA accumulation in the cytoplasm without affecting the formation and nuclear entry of the viral DNA [1]. Further studies demonstrate that ZAP directly binds to specific viral mRNAs [3], [4], [5], recruits polyA ribonuclease (PARN) to shorten the polyA tail [4], and recruits the RNA exosome to degrade the RNA body from the 3′ end [4], [6]. In addition, ZAP recruits the cellular decapping complex to initiate degradation of the target viral mRNA from the 5′ end [4]. The DEAD-box RNA helicase p72 directly interacts with ZAP and is required for optimal function of ZAP [7].

Whether a virus is sensitive to ZAP seems to be determined by the presence of ZAP-responsive element (ZRE) in the viral mRNA. The ZRE in MoMLV was mapped to the 3′UTR and the ZREs in SINV were mapped to multiple fragments [5]. For Ebola virus and Marburg virus the ZRE was mapped to the L domain [3], and the ZREs of HIV-1 were mapped to the 5′UTRs of multiply spliced mRNAs [4]. The only common feature of these ZAP target sequences is that they are all more than 500 nt long; no obvious common sequence or motifs can be identified in these ZREs. Thus whether a virus is susceptible to ZAP can only be determined experimentally.

Xenotropic murine leukemia virus-related virus (XMRV), a γ-retrovirus, was originally thought to be involved in prostate cancer in a cohort of patients lacking a functional RNaseL gene [8]. However, in the follow up studies, little or no XMRV was detected in patients with prostate cancer, raising questions on XMRV's role in prostate cancer [9], [10], [11], [12]. XMRV was also thought to be involved in chronic fatigue syndrome (CFS) [13]. Subsequent analyses by laboratories from many countries, however, reported the absence of XMRV infection in CFS patients [14], [15], [16], [17], and re-examinations of samples from patients previously identified as XMRV-positive in the original study found no consistent evidence of XMRV infection [17], [18]. These results provoked serious doubt on the relationship between XMRV and human diseases. In late 2011, strong evidence was provided that the virus was just a laboratory artefact generated by recombination of two mouse viruses during passage of a human prostate-tumour xenograft [19]. Detection of the virus in patient samples is likely laboratory contamination with XMRV produced by a prostate cancer cell line or with other commercial laboratory reagents [17], [19]. Nonetheless, XMRV has been extensively studied as a new retrovirus [20], [21], [22], [23], [24]. Comparison of XMRV with related retroviruses provides insights into the detailed mechanisms for retroviral replication.

In this report we show that human ZAP inhibits XMRV infection by preventing the accumulation of viral mRNA in the cytoplasm.

## Results

### 1. Overexpression of hZAP inhibits XMRV infection

**Figure 1:**
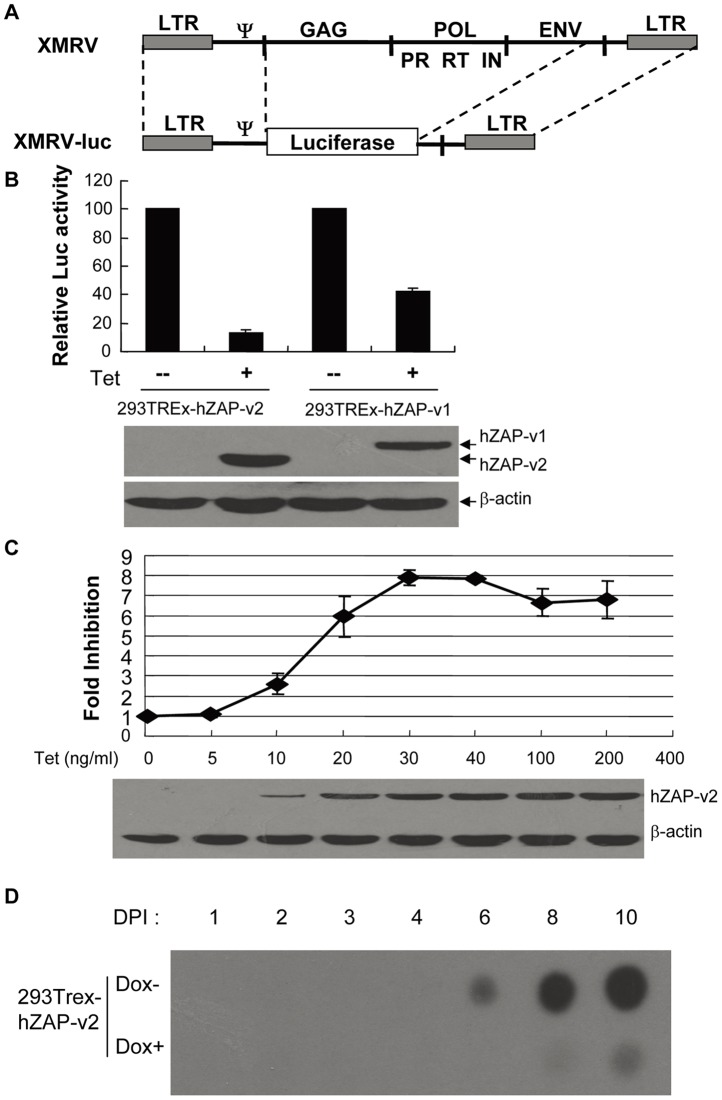
Overexpression of hZAP inhibits XMRV infection. (A) Schematic structure of XMRV-luc vector. The coding sequences of Gag-Pol and part of Envelope were replaced with luciferase-coding sequence to generate pXMRV-luc. (B) Overexpression of hZAP inhibits XMRV-luc infection. 293TRex cells expressing hZAP-v1-myc and hZAP-v2-myc upon tetracycline induction were infected with VSV-G pseudotyped XMRV-luc. Cells were equally divided into two dishes at 6 h postinfection, with one mock treated and the other treated with tetracycline. Cells were lysed and luciferase activity was measured at 48 h postinfection (upper panel). The luciferase activity in the absence of ZAP was set as 100. Data presented are means ± SD of three independent experiments. The expression of hZAP was confirmed by Western blotting (lower panel). (C) ZAP inhibits XMRV-luc in an expression-level-dependent manner. 293TREx-hZAP-v2 cells were infected with XMRV-luc. At 6 h postinfection the cells were equally split and tetracycline was added to the concentrations indicated. Cells were lysed and luciferase activity was measured at 48 h postinfection. Fold inhibition was calculated as the luciferase activity in mock treated cells divided by the luciferase activity in the tetracycline treated cells (upper panel). Data presented are means ± SD of three independent experiments. The expression levels of hZAP-v2 were measured by Western blotting (lower panel). (D) ZAP inhibits XMRV replication. 293Trex-hZAP-v2 Cells were infected with XMRV produced in 293T cells. At 8 h postinfection, cells were mock treated or treated with doxycycline to induce hZAP-v2 expression. Samples were taken every day and subjected to RT assays.

Due to the similarity between XMRV and MoMLV, we speculated that ZAP might inhibit XMRV by the same mechanism as it inhibits MoMLV. To facilitate sample handling and detection of viral infection, we constructed an XMRV vector carrying the firefly luciferase reporter gene. Since ZAP inhibits the expression of MLV-luc vector, the XMRV reporter was generated in a similar manner as constructing MLV-luc [5]. The coding sequences of Gag-Pol and part of the envelope protein of XMRV were replaced with the luciferase coding sequence to generate pXMRV-luc (Fig. 1A). XMRV-luc pseudovirus was produced by cotransfecting pXMRV-luc with plasmids expressing VSVG and MLV Gag-pol into HEK 293T cells.

There are two forms of human ZAP (hZAP) arising from alternative splicing, which differ only at the C-terminal domain [25]. Myc-tagged full-length ZAP (hZAP-v1) and the short form (hZAP-v2) were expressed in HEK293 cells in a tetracycline-inducible manner. To test whether XMRV is sensitive to ZAP, the cells were challenged with XMRV-luc and assayed for luciferase expression with or without ZAP expression. The expression of both hZAP-v1 and hZAP-v2 inhibited the expression of XMRV-luc (Fig. 1B).

To assess whether ZAP's inhibitory effect on XMRV-luc is dependent on the expression level of ZAP, hZAP-v2 expression was induced by increasing concentrations of tetracycline. With the increasing expression level of hZAP-v2, fold inhibition of hZAP-v2 against XMRV-luc increased accordingly (Fig. 1C), indicating the antiviral activity of hZAP-v2 against XMRV-luc is dependent on the expression level of ZAP.

To test whether ZAP is able to inhibit the replication of XMRV, replication-competent virus was produced by transfecting XMRV proviral DNA into HEK 293T cells. 293Trex-hZAP-v2 cells were infected with XMRV, followed by treatment of the cells with doxycycline to induce ZAP expression. Virus spreading was monitored by measuring reverse transcriptase (RT) activity in the cell culture supernatants. In the absence of ZAP expression, the peak RT activity was detected at 10 days postinfection (Fig. 1D), which is consistent with the report that XMRV replicates relatively poorly in HEK 293T cells [20]. In contrast, when ZAP expression was induced, only very weak signal was detected (Fig. 1D). These results indicate that ZAP inhibits the propagation of replication-competent XMRV.

### 2. Downregulation of endogenous hZAP enhances XMRV-luc infection

**Figure 2:**
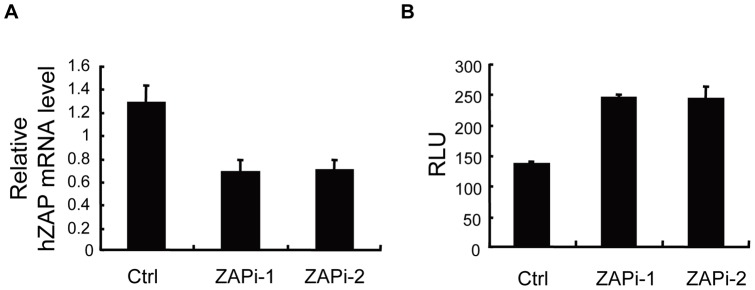
Downregulation of endogenous hZAP enhances XMRV-luc expression. HOS cells were transfected with control siRNA (Ctrl) or siRNAs directed against hZAP (ZAPi-1 and ZAPi-2), followed by infection with XMRV-luc for 5 h. At 48 h postinfection, cells were lysed. (A) Endogenous hZAP mRNA levels were measured by real-time PCR. (B) Luciferase activity was measured and presented as relative light units (RLU). Data presented are means ± SD of three measurements.

To test whether endogenous hZAP inhibits XMRV-luc, HOS cells were transfected with siRNAs directed against hZAP (ZAPi-1 and ZAPi-2) to downregulate endogenous ZAP expression, and then challenged with XMRV-luc. The ZAP mRNA levels were downregulated by about 60% (Fig. 2A). As expected, the expression of XMRV-luc was significantly increased in cells transfected with the siRNAs directed against ZAP compared with control cells (Fig. 2B), indicating that endogenous hZAP was active against XMRV-luc infection.

### 3. Expression of hZAP prevents the accumulation of XMRV-luc mRNA

ZAP has been demonstrated to inhibit MoMLV infection by promoting viral mRNA degradation in the cytoplasm without affecting the formation and nuclear entry of the viral DNA [1]. To confirm that ZAP inhibits XMRV by the same mechanism, 293TRex-hZAP-v2 cells were infected with XMRV-luc at different dilutions, and the nuclear circular viral DNA was analyzed by PCR amplification. As expected, comparable levels of the nuclear circular DNA were detected before and after induction of hZAP expression (Fig. S1).

**Figure 3:**
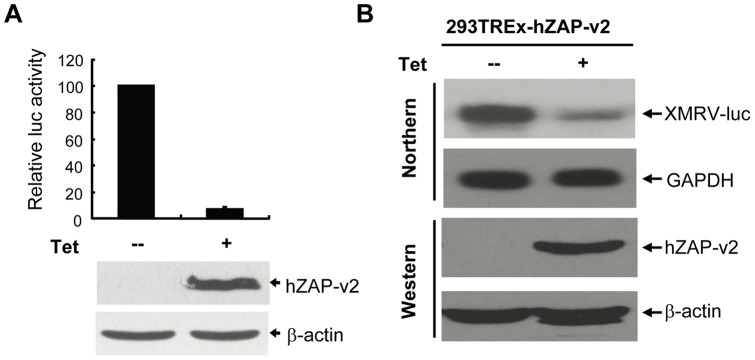
Expression of hZAP prevents the accumulation of XMRV-luc mRNA. 293TRex-hZAP-v2 cells harbouring XMRV-luc provirus were mock treated or treated with 1μg/ml tetracycline for 48 h to induce ZAP expression. (A) Cells were lysed and luciferase activity was measured (upper panel). Data presented are means ± SD of three independent experiments. The expression of hZAP was confirmed by Western blotting (lower panel). (B) Cytoplasmic RNA was extracted and subjected to Northern blotting to detect the mRNA indicated (upper panel). Expression of hZAP was confirmed by Western blotting (lower panel).

To analyze whether hZAP promotes XMRV mRNA degradation, 293Trex-hZAP-v2 cells were infected with XMRV-luc and cultured for an extensive period of time to establish stable infection. ZAP expression significantly inhibited XMRV-luc expression (Fig. 3A). Consistently, XMRV-luc mRNA levels were significantly reduced after the induction of ZAP expression, as measured by Northern blotting (Fig. 3B).

**Figure 4:**
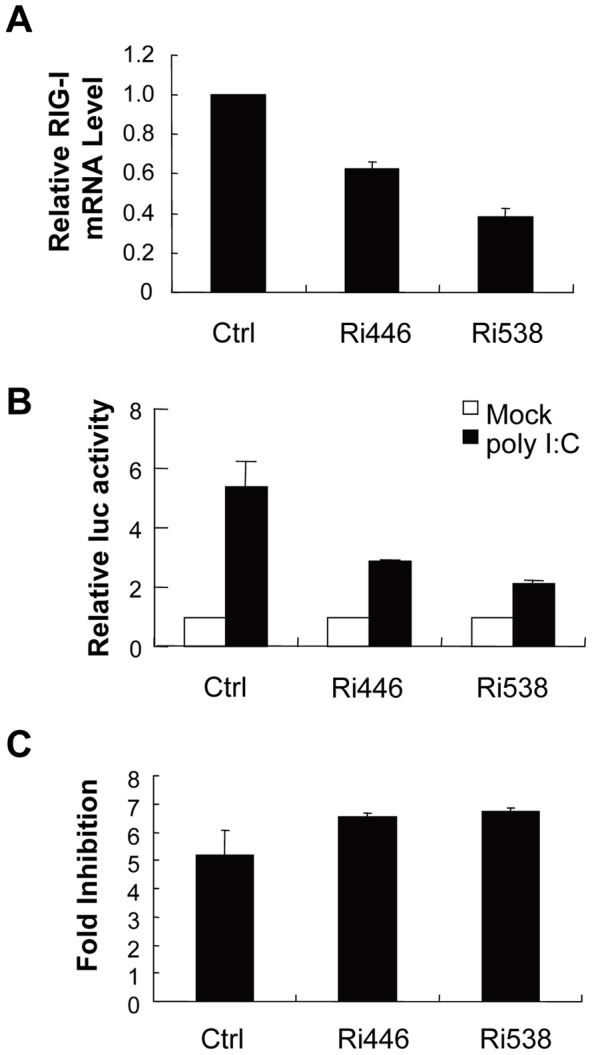
Downregulation of RIG-I does not affect ZAP's antiviral activity against XMRV-luc. (A) Control shRNA and shRNAs against RIG-I (Ri446 and Ri583) were stably expressed in 293TRex-hZAP-v2 cells. RIG-I mRNA levels were measured by real-time PCR and normalized to that of GAPDH. Data presented are means ± SE of three parallel experiments. (B) Cells were transfected with pGl3-IFNβ-luc and pRL-TK. At 48 h posttransfection, cells were transfected with poly (I:C). Luciferase activity was assayed 12 h later. (C) Cells were infected with XMRV-luc, mock treated or treated with tetracycline for 48 h to induce ZAP expression, and luciferase activities were measured. Fold inhibition was calculated as the luciferase activity in mock treated cells divided by that in tetracycline treated cells. Data presented are means ± SE of three parallel experiments.

ZAPs was recently reported to stimulate type I interferon production through interaction with RIG-I [26]. To explore whether ZAP inhibits XMRV infection by activating the RIG-I pathway, endogenous RIG-I expression was downregulated in 293Trex-hZAP-v2 cells by RNAi (Fig. 4A). Downregulation of RIG-I impaired poly (I:C)-activated IFNβ-luc reporter expression (Fig. 4B), but had little effect on the antiviral activity of ZAP against XMRV (Fig. 4C), implicating that inhibition of XMRV infection by ZAP is independent of the RIG-I pathway.

### 4. ZAP targets 3′UTR of XMRV

**Figure 5:**
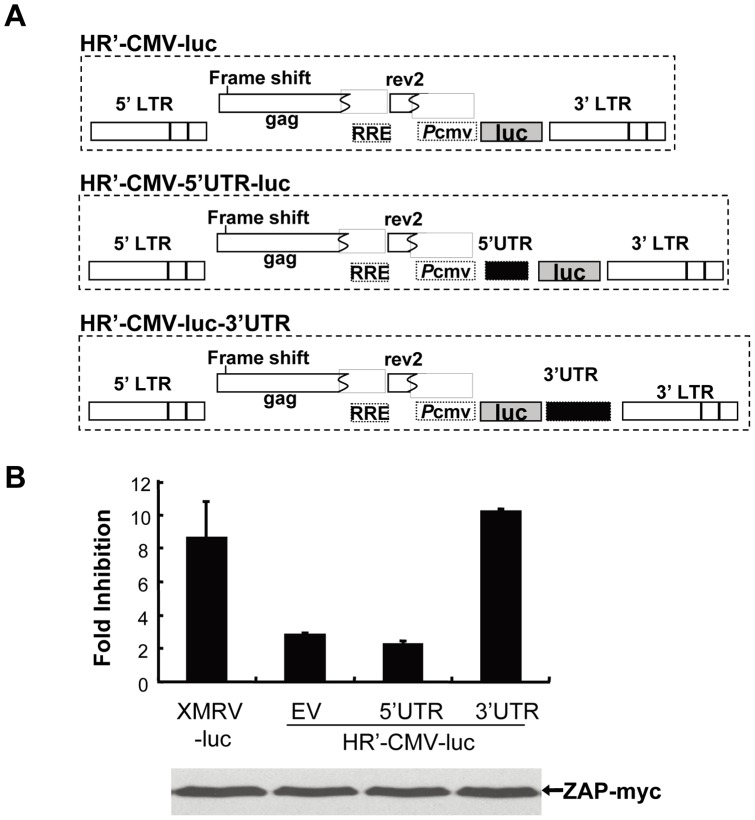
ZAP targets the 3′LTR of XMRV. (A) Schematic structures of HR'-CMV-luc vectors. (B) 293TRex-hZAP-v2 cells were infected with the vectors indicated. At 3 h postinfection, cells were mock treated or treated with 1 μg/ml tetracycline to induce ZAP expression. At 48 h postinfection the cells were lysed and luciferase activity was measured. Fold inhibition was calculated as luciferase activity in mock treated cells divided by luciferase activity in tetracycline treated cells (up panel). Data presented are means ± SD of three independent experiments. The expression of hZAP-v2 was confirmed by Western blotting (lower panel).

Previous studies demonstrate that ZAP targets specific viral mRNA sequences [5]. To identify the ZAP-responsive element (ZRE) in XMRV, the sequence corresponding to the 5′ or 3′ UTR of XMRV-luc was cloned into pHR'-CMV-luc (Fig. 5A), a lentivector that is not responsive to ZAP [4]. The vectors were packaged to infect 293TRex-hZAP-v2 cells and assayed for their sensitivities to ZAP. The vector containing the 3′UTR sequence displayed sensitivity comparable to that of XMRV-luc. In contrast, the vector containing the 5′UTR failed to do so (Fig. 5B). These results established that the 3′UTR of XMRV-luc is the target sequence of ZAP.

**Figure 6:**
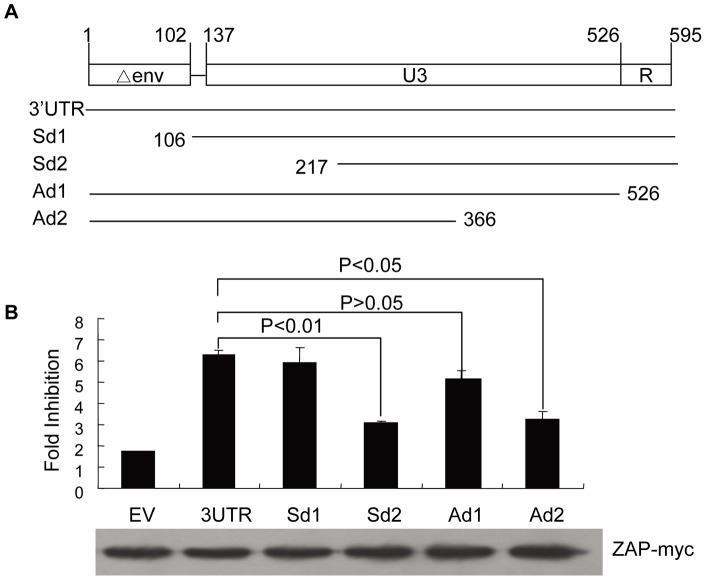
Mapping of ZRE in the 3′ LTR of XMRV. (A) Schematic structures of the truncation constructs of 3′UTR. (B) Analysis of the sensitivity of the 3′UTR truncation mutants to ZAP. Fold inhibition was measured as described in the legend to Figure 5B. Data presented are means ± SD of three independent experiments.

The ZRE in MoMLV was also mapped to the 3′UTR [5]. Sequence analysis reveals that the 3′UTRs of XMRV and MoMLV share 67.9% identity (Fig. S2). To map the minimal sequence required for response to ZAP, the 3′UTR of XMRV was truncated and the truncation mutants were analyzed for their sensitivity to ZAP (Fig. 6A). The above identified XMRV 3′UTR covers a short fragment of env, U3 and the R region. Deletion of the env sequence and R region did not significantly affect the sensitivity to ZAP (Fig. 6B). However, further deletion resulted in a significant drop in the sensitivity (Fig. 6B), suggesting that the fragment covering the U3 region is the ZRE in XMRV.

## Discussion

Infection of cells by retroviruses can be restricted by host factors through a variety of mechanisms [27]. APOBEC3G and some of its family members primarily target the single-stranded viral DNA generated during reverse transcription [28]. TRIM5α targets HIV-1 capsid (CA) and thus blocks viral infection in the early phase [29]. Friend-virus susceptibility factor 1 (Fv1) also inactivates the incoming viral capsids after entry [30]. Tetherin inhibits the release of HIV-1 particles from the infected host cells by “tethering” the nascent retroviral particles to the plasma membrane [31]. Recently, several restriction factors have been demonstrated to significantly inhibit the replication of XMRV, including APOBEC3, Fv1 and Tetherin [21]. However Trim5α, which inhibits N-tropic MLV, failed to inhibit XMRV infection [21]. Hence, whether XMRV is restricted by a given factor seems to require experimental determination.

ZAP has been reported to inhibit the infection of MoMLV, HIV-1, Ebola virus, Marburg virus and certain alphaviruses [1], [2], [3], [4]. In the present study, we report that ZAP inhibits the expression of XMRV vector and the propagation of replication-competent XMRV. Consistent with the previously reported mechanisms by which ZAP inhibits viral infection, ZAP significantly inhibited the accumulation of XMRV-luc mRNA in the cytoplasm.

ZAP binds directly to ZRE-containing viral mRNAs. No obvious common motifs or secondary structures have been observed in the so far identified ZREs. The crystal structure of the N-terminal domain of ZAP, the putative major RNA-binding domain, predicts that the target RNA should have a tertiary structure to place some nucleotides in the correct position to fit into a three-dimensional cleft on ZAP surface [32]. Furthermore, two ZAP-binding modules are required for a ZAP-responsive RNA [32]. The ZAP responsive element (ZRE) in XMRV mRNA was mapped to the 3′UTR (Fig. 5A, B). Further mapping results suggest that the ZRE of XMRV is in the 420bp fragment covering the U3 region (Fig. 6B). Comparison of the 3′UTRs of XMRV and MoMLV provides some information about the sequences required for the RNA to be responsive to ZAP (Fig. S2). For example, the majority of the enhancer 1 region of MoMLV is missing in XMRV, suggesting that this region is not required for binding to ZAP. Furthermore, sequence comparison of the 3′UTRs of MoMLV, XMRV, ecotropic MLV, amphotropic MLV, xenotropic MLV and polytropic MLV reveals that the 3′UTRs of amphotropic MLV and ecotropic MLV share more than 80% identity to that of MoMLV and that the 3′UTRs of xenotropic MLVs and polytropic MLV shared more than 85% identity to that of XMRV. Thus, we speculate that these MLVs may all be sensitive to ZAP.

In summary, here we report that ZAP inhibits XMRV infection by targeting the viral mRNA for degradation in the cytoplasm. The ZRE in XMRV is mapped to the U3 region of the 3′UTR. Such findings broaden the antiviral spectrum of ZAP.

## Materials and Methods

### Plasmid construction

pCR2-TOPO-VP62, an infectious clone of XMRV was kindly provided by Dr. Stephen P. Goff (Columbia University, Howard Hughes Medical Institute) [20]. pXMRV-luc is an XMRV vector carrying 5′ LTR of XMRV, luciferase coding sequence and 3′LTR of XMRV. The 5′LTR of XMRV was PCR-amplified with forward primer 5LTRup bearing an *Eco*RI site and reverse primer 5LTRdown bearing a *Bam*HI site. To generate the 3′LTR of XMRV, a PCR fragment generated using primers 3LTRup and 3LTRm3 was mixed with a PCR fragment generated using primers 3LTRm5 and 3LTRdown, and PCR-amplified using primers 3LTRup bearing a *Not*I site and 3LTRdown. The luciferase-coding sequence was amplified from pMLV-luc [1] with forward primer X-luc5 bearing a BamHI site and reverse primer X-luc3 bearing a *Not*I site. To generate pXMRV-luc, the PCR fragment of 3′LTR was cloned into pMD18-T vector (TaKaRa), followed by cloning the 5′ LTR fragment upstream of the 3′LTR. The PCR-derived coding sequence of luciferase was inserted between 5′LTR and 3′LTR. Sequences of the primers are listed below with restriction sites capitalized:

5LTRup: 5′-gGAATTCgctgaaagaccccaccataag;

5LTRdown: 5′-cgGGATCCgtccctagatctcgagaacactt;

3LTRup: 5′-aGCGGCCGCtttgtaaaagacagaatttcg;

3LTRm3: 5′-gttgttagtttcgctttatctgagg;

3LTRm5: 5′-cctcagataaagcgaaactaacaac;

3LTRdown: 5′-caaatgaaagacccccgagctgggtag;

X-luc5: 5′-cgGGATCCaccatggaagacgccaaaaacat;

X-luc3: 5′-ccgcgtGCGGCCGCttacaatttggactttcc

pHR'-CMV-Luc, a lentivector that is not sensitive to ZAP, has been described previously [33]. Plasmid pHR'-CMV-MCS-Luc was modified from pHR'-CMV-Luc by deleting the *Bam*HI site, followed by inserting a fragment containing *Bam*HI, *Eco*RI and *Sal*I sites between the CMV promoter and luciferase-coding sequence. To generate pHR'-CMV-5′ UTR-luc, the 5′ UTR of XMRV was PCR-amplified with forward primer UTR5-5 bearing an *Eco*RI site and reverse primer UTR5-3 bearing a *Sal*I site and cloned into pHR'-CMV-MCS-Luc. To generate pHR'-CMV-luc-3′UTR, a fragment covering the luciferase-coding sequence and 3′UTR of XMRV was amplified from pXMRV-luc with primers Luc3-5 bearing a *Bam*HI site and UTR3-3 bearing an *Xho*I site to replace the *Bam*HI-*Xho*I fragment of pHR'-CMV-MCS-Luc. The 3′UTR truncation mutants were generated by PCR-amplification of desired sequences with primers bearing an *Xho*I site upstream of the matching sequences. The resulting fragments were inserted into the *Xho*I site in pHR'-CMV-MCS-Luc. Sequences of the primers are listed below with restriction sites capitalized.

UTR5-5: 5′-aaGAATTCagccttttgctgtttgcatc

UTR5-3: 5′-ttcaGTCGACggatccgtccctagatct

Luc3-5: 5′-ggacggatccaccatggaagacgccaa

UTR3-3: 5′-ccagCTCGAGtgggaacacgggtacccg

Sd1-F: 5′- agCCGCTCGAGattttattcagtttc

Sd2-F: 5′-agCCGCTCGAGttctcaaaagttacaag

Sd-R: 5′-ccagCTCGAGtgggaacacgggtacccg

Ad-F: 5′-agCCGCTCGAGtgtaaaagacagaatttc

Ad1-R: 5′-agCCGCTCGAGgccgagtgtggagttc

AD2-R: 5′-agCCGCTCGAGaaactgttgttagt

The plasmids expressing shRNAs directed against RIG-I (Ri446 and Ri538) and a control shRNA were generated by annealing pairs of oligonucleotides and cloning into pSuper-retro-puro (OligoEngine) using *Bgl*II and *HindIII* sites following the manufacture's instructions. The target sequences are listed below.

Control: 5′-GCAAGCTGACCCTGAAG

shRIGi446: 5′-CCATGTGAAGTACAAGACA

shRIGi538: 5′-GCAAGATCTTACTCAGAGA

pcDNA4TO/myc-hZAP-v1 and pcDNA4TO/myc-hZAP-v2, which express myc-tagged hZAP-v1 and hZAP-v2, respectively, have been describes previously [4].

### siRNA and siRNA transfection

Control siRNA (siCtrl: Catalog No. D-001810-10) and siRNAs against hZAP (ZAPi-1: Catalog No. J-017449-11 and siZAPi-2: Catalog No. J-017449-09) were obtained from Thermo Scientific. siRNA was transfected into cells by lipofectamine 2000 (Invitrogen) following the manufacturer's protocol.

### Cells and viral infection

All the cells were maintained in DMEM supplemented with10% FBS. Transfection was performed using lipofectimine 2000 (Invitrogen) following the manufacturer's instruction. 293TRex-hZAP-v1 and 293TRex-hZAP-v2 cells have been described previously [4].

Production of VSV-G pseudotyped SR-Ctrl, SR-RIG-Ii446 and SR-RIG-Ii583 transducing viruses, and transduction of 293TRex-hZAP-v2 cells with these viruses have been previously reported [4].

VSV-G pseudotyped XMRV-luc was produced by cotransfection of 293T cells with pVSV-G, pHIT60 and the XMRV-luc vector. To produce VSV-G pseudotyped lentiviruses, 293T cells were cotransfected with pVSV-G and pCMVdelR8.2 (a plasmid expressing HIV Gag and Pol proteins), and pHR'-CMV-mcs-luc vectors.

Replication-competent XMRV was produced by transfection of 293T cells with pCR2-TOPO-VP62. Plasmid pVSVG was included to enhance XMRV infection efficiency.

To evaluate the antiviral activity of ZAP, cells were infected with XMRV-luc or HR'-CMV-mcs-luc based vectors. At 5 h postinfection, cells were equally divided into two dishes, with one mock treated and the other treated with tetracycline. The cells were lysed and luciferase activities were measured with the Luciferase Assay System (Promega) at 48 h postinfection. Fold inhibition was calculated as the luciferase activity in mock treated cells divided by that in tetracycline treated cells.

To establish a 293Trex-hZAP-v2 cell line carrying XMRV-luc provirus, 293Trex-hZAP-v2 cells were infected for 4 times with XMRV-luc, followed by cultivation and passage for a week.

### Hirt DNA extraction and detection of nuclear circular viral DNA

293TRex-hZAP-v2 cells were seeded in 35 mm dishes and infected with XMRV-luc viruses at varying dilution on the next day. Right after infection the cells were untreated or treated with tetracycline to induce hZAP-v2 expression. At 24 h postinfection, Hirt DNA was extracted as described previously [34], and the nuclear circular DNA was detected by PCR-amplification of the 2-LTR junction using primers X2LTR5 and X2LTR5. Mitochondrion DNA (mtDNA) amplified with primers hmtDNAsp and hmtDNAap were used as an internal control. PCR conditions were 94°C for 30 s, 57°C for 30 s, and 72°C for 45 s for 40 cycles. Sequences of the primers are listed below.

X2LTR5: 5′-agtcatccgatagactgag

X2LTR3: 5′-ttatagggctaggactggg

hMtDNAsp: 5′-GACGTTAGGTCAAGGTGTAG

hMtDNAap: 5′-GGTTGTCTGGTAGTAAGGTG

### Real-time PCR

Cytoplasmic RNA was extracted using RNeasy Kit (Qiagen) following the manufacturer's instruction, followed by reverse transcription with MLV reverse transcriptase using random primers. The mRNA levels of hZAP and RIG-I were measured by SYBR Green real-time PCR in Rotor-gene 6000 (Corbett Life Science) using the following program: 1) 50°C 2 min, 1 cycle; 2) 95°C 5 min, 1 cycle; 3) 95°C 15 s -> 60°C 30 s -> 72°C 30 s, 40 cycles; 4) 72°C 10 min, 1 cycle. The mRNA level of *gapdh* served as the internal control. The sequences of the primers are listed below:

qhZAP FP: CCACATCTTCTAGGGTGGATGA

qhZAP RP: CGTCCAGGTTTTACCAATAAGCA

qRIG-I FP: CCTACCTACATCCTGAGCTACAT

qRIG-I RP: TCTAGGGCATCCAAAAAGCCA

qGAPDH FP: ATGGGGAAGGTGAAGGTCG

qGAPDH RP:GGGGTCATTGATGGCAACAATA

### XMRV spreading assay

293Trex-hZAP-v2 Cells were seeded in 60 mm disks and infected the day after with 2 ml XMRV virus produced in 293T cells. At 8 h postinfection, cells were mock treated or treated with doxycycline to induce expression of hZAP-v2. Supernatants were collected every day and subjected to RT assays as described previously [35].

### Northern blotting

Cytoplasmic RNA was isolated from cells with an RNeasy kit (Qiagen) according to the manufacturer's instructions. The RNA samples were separated by electrophoresis, transferred to nylon membrane, and hybridized for 15–20 h with 32P-labeled probes prepared by a random primer labeling kit (Stratagene, La Jolla, CA). The probe for XMRV-luc mRNA was the coding sequence of *firefly* luciferase. The probe for *gapdh* mRNA was the coding sequence of *gapdh*. The nylon membrane was washed three times with 0.1×SSC (pH 7) and 0.1% SDS at 65°C and exposed to x-ray film.

### Poly**(**I:C**)** stimulation assay

293TRex-hZAP-v2-Ctrl, 293TRex-hZAP-v2-Ri446, and 293TRex-hZAP-v2-Ri583 cells (∼1×10^5^) were seeded on 24-well plates and transfected with reporter plasmid pGL3-IFNβ-luc (a kind gift from Prof. Zhengfan Jiang, Peking University, China) on the following day. To normalize transfection efficiency, 0.01 μg of pRL-TK Renilla reporter plasmid (Promega) was included in each transfection. Cells were mock treated or treated with tetracycline (1 μg/ml) to induce ZAP expression. At 48 h posttransfection, cells were stimulated for 12 h by transfection of 0.25 μg poly (I:C), and luciferase activity was measured with the Dual-Luciferase Reporter Assay system (Promega).

## Supporting Information

Figure S1 **hZAP does not block the formation and nuclear entry of XMRV-luc proviral DNA.** 293TREx-hZAP-v2 cells were infected with XMRV-luc virus at the indicated dilutions. At 6 h postinfection, cells were mock treated or treated with 1 μg/ml tetracycline. At 24 h postinfection, cells were lysed and Hirt DNA was extracted. The 2-LTR junction of the nuclear circular viral DNA was detected by PCR. PCR product of mitochondrion DNA (mtDNA) was used as an internal control. The data is representative of three independent experiments.
(TIF)

Figure S2 **Sequence comparison of the 3**′ **UTRs of different MLVs.** The 3′UTRs of XMRV, MoMLV (AF033811), Friend MLV (NC_001362), amphotropic MLV 1313 (AF411814), xenotropic MLV LAPC4 (JF908816), xenotropic MLV DG-75 (AF221065), polytropic MLV MCF 1233 (U13766) were aligned using Vector NTI 10.0.1. X, xenotropic; P, polytropic; A, amphotropic; E, ecotropic.
(TIF)
