# Supplementary figures and images for: Zinc-Finger Antiviral Protein Inhibits XMRV Infection
Source: PLoS One. 2012 Jun 15;7(6):e39159. doi: 10.1371/journal.pone.0039159 (PMC3376128; doi:10.1371/journal.pone.0039159)

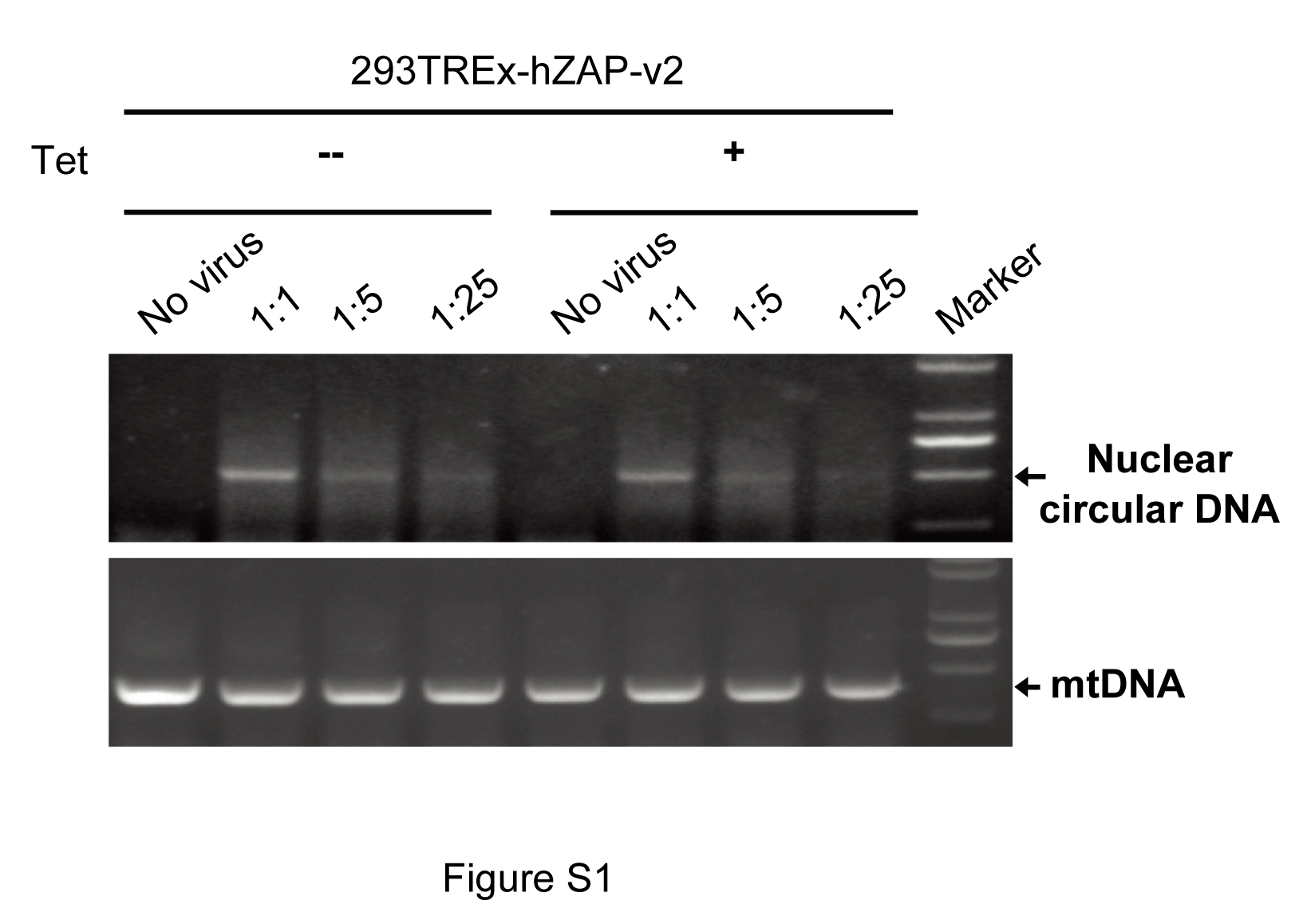

Supplement: Figure S1 — hZAP does not block the formation and nuclear entry of XMRV-luc proviral DNA. 293TREx-hZAP-v2 cells were infected with XMRV-luc virus at the indicated dilutions. At 6 h postinfection, cells were mock treated or treated with 1 μg/ml tetracycline. At 24 h postinfection, cells were lysed and Hirt DNA was extracted. The 2-LTR junction of the nuclear circular viral DNA was detected by PCR. PCR product of mitochondrion DNA (mtDNA) was used as an internal control. The data is representative of three independent experiments. (TIF) [file pone.0039159.s001.tif]

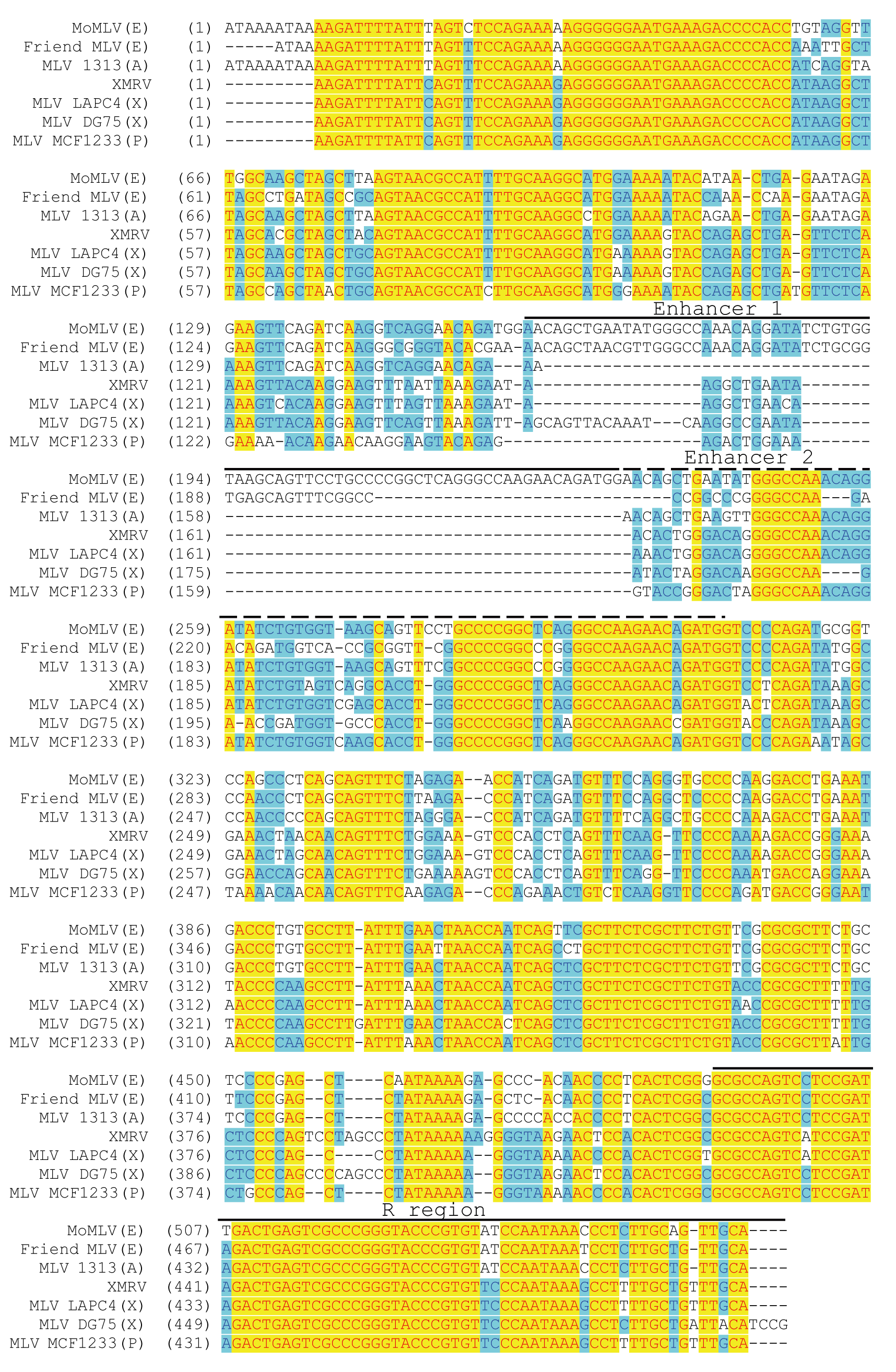

Supplement: Figure S2 — Sequence comparison of the 3′ UTRs of different MLVs. The 3′UTRs of XMRV, MoMLV (AF033811), Friend MLV (NC_001362), amphotropic MLV 1313 (AF411814), xenotropic MLV LAPC4 (JF908816), xenotropic MLV DG-75 (AF221065), polytropic MLV MCF 1233 (U13766) were aligned using Vector NTI 10.0.1. X, xenotropic; P, polytropic; A, amphotropic; E, ecotropic. (TIF) [file pone.0039159.s002.tif]
